# Effects of NDRG2 Overexpression on Metastatic Behaviors of HCT116 Colorectal Cancer Cell Line

**DOI:** 10.15171/apb.2017.080

**Published:** 2017-12-31

**Authors:** Ali Golestan, Abbas Ghaderi, Zahra Mojtahedi

**Affiliations:** ^1^Autophagy Research Center, Shiraz University of Medical Sciences, Shiraz, Iran.; ^2^Institute for Cancer Research, Shiraz University of Medical Sciences, Shiraz, Iran.

**Keywords:** Colorectal cancer, NDRG2, Invasion, Migration, Matrix metalloproteinase

## Abstract

***Purpose:*** N-myc downstream-regulated gene 2 (NDRG2) is frequently down-regulated in cancer, and plays an important role in the control of tumor growth and metastasis. Its manipulation has been suggested as a therapy in cancer. Here, we examined the outcome of NDRG2 overexpression on proliferation, invasion, migration and MMP activity of HCT116 colorectal cancer cell line.

***Methods:*** The HCT116 cell line (human colorectal cancer) was transfected with pCMV6-AC-GFP-NDRG2. 2,5diphenyltetrazolium bromide (MTT) assay was used to detect cell proliferation. The invasion and migration of the transfected cells were examined through transwell chambers while the MMP-9 activity was detected by the ability of the cells to digest gelatin.

***Results:*** Overexpression of NDRG2 by stable NDRG2 transfection decreased cell proliferation, migration and invasion ability, along with decreasing MMP-9 activity.

***Conclusion:*** Our data indicate that NDRG2 overexpression can suppress several aspect of tumorigenesis. Further investigations are necessitated to verify if NDRG2 molecule can be a therapeutic target in colorectal cancer.

## Introduction

Colorectal cancer is a top-cancer killer worldwide, and its incidence is rising in several countries including Iran.^1^ Novel therapeutic targets remain the choice to overcome the advanced disease and treatment failure. N-myc downstream-regulated gene 2 (NDRG2) belongs to a family of differentiation-related molecules.^2^ NDRG2 has shown tumor suppressor activities in certain cancer models, and its manipulation has attracted much attention as a therapeutic target in cancer.^3^

NDRG2 is mainly expressed in skeletal muscles, the heart, brain, liver and kidney under physiological conditions.^4^ It is reported that NDRG2 is under expressed in certain types of cancer (e.g., liver, thyroid, and breast cancers), suggesting a possible role for NDRG2 in tumor suppression,^5,6^ In vitro assays in several cancers such as colon have shown that tumor migration and invasion were diminished after NDRG2 overexpression.^7^ An important mechanism which links the overexpression of NDRG2 to compromising invasion is the downregulation of matrix metalloproteinases (MMPs), a group of zinc-dependent endo-proteinases with remarkable roles in tissue remodeling and homeostasis.^3,8^ MMPs are main culprit molecules in advancing invasion and metastasis through digestion of extracellular matrix (ECM) and basement membrane components^3^. In addition to invasion ability, NRGD2 overexpression has been demonstrated to inhibit cell proliferation and upregulate tumpr suppressor p53.^9^ These findings point out NDRG2 as a potential tumor suppressor gene in the studied models.

Emerging evidence demonstrates the involvement of NDRG2 in colon cancer. In recent studies, NDRG2 was found to decrease in colorectal carcinoma.^10^ Furthermore, NDRG2 expression inhibited colon cancer cell proliferation by down regulation of AP-1 activity.^11^ Whether NDRG2 expression plays different roles in different types of colon cancer needs more clarification.

Mutation in the Kirsten Ras (KRAS) oncogene occurs in up to 30% of colorectal cancer patients which has been associated with poorer survival.^12^ We have previously investigated how NDRG2 overexpression affects cell proliferation and invasion in SW48 cells,^13^ a highly invasive colon cell line with no KRAS mutation.^14^ We found that overexpression of NDRG2 reduced SW48 cell proliferation as well as migration and invasion, possibly through suppressing MMP-9 activity. Here, we investigated the outcome of NDRG2 overexpression on proliferation, invasion, migration, and MMP-9 activity in HCT116 cells, a model of colon cancer with a KRAS mutation.

## Materials and Methods

The human colorectal cancer cell line HCT116 (ATCC^R^ CCL-231^TM^) was purchased from Pasteur institute of Iran. Cells were grown under standard conditions.^13^ Shuttle vector pCMV6-AC-GFP plasmid (Origene), with and without NDRG2 gene, was amplified with manual amplification protocol as described previously.^13^ E.coli competent cells were prepared and transformed with the plasmid, then plasmid was purified using PureLink™ HiPure Plasmid Filter Purification Kit (Invitrogen). Lipofectamine (Invitrogen) was used to transfect HCT116 cells with the plasmid pCMV6-AC- GFP-NDRG2 (NDRG2 group) or pCMV6-AC-GFP vector alone (mock group).

Stable transfected HCT116 cells were produced. Cell growth following stable transfection was evaluated by MTT assay. The ability of cells for invasion and migration was measured through the number of cells penetrating transwell membrane of Boyden chambers. In vitro wound healing was analyzed using scratch assay. The activity of MMP-9 was detected using gelatin zymography. These assays were applied to each of the three groups: WT group (non infected cells), mock group (pCMV6-AC-GFP) and NDRG2 group (pCMV6-AC-GFP-NDRG2). The details of all the above experiments were previously described.^13^

Statistical analysis was done using Graphpad Prism (version5) software. One-way ANOVA calculated the differences between studied groups followed by Tukey Post Hoc multiple comparisons. For wound healing assay, student *t*-test was performed. All data are represented by the mean ± standard deviation of three independent experiments. A P value less than 0.05 was considered significant.

## Results and Discussion

Here, we explored the effect of overexpression of NDRG2, a potential tumor suppressor molecule, on proliferation, migration and invasion ability of HCT116 colon cancer cell line. HCT116cells were stably transfected with either pCMV6-AC-GFP (mock) or pCMV6-AC-GFP-NDRG2 plasmid.

**Figure 1:**
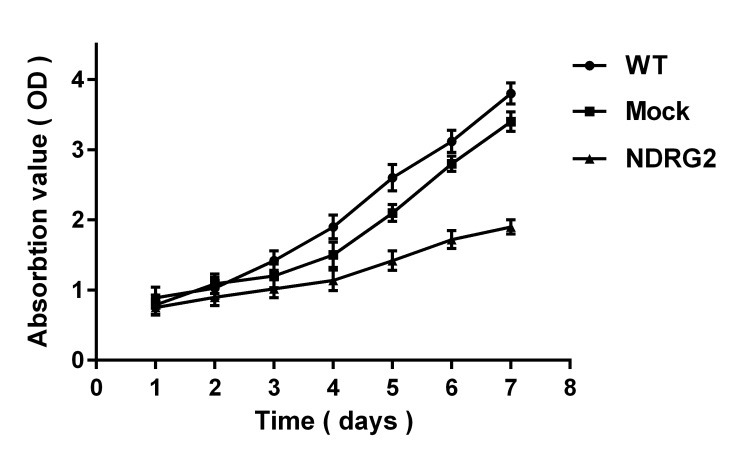
Overexpression of NDRG2 inhibits HCT116 cell proliferation. Cell proliferation rate was determined by MTT assay. The assays were repeated thrice, and illustrated as the mean ± SD. *=P<0.001.

In order to investigate the effect of NDRG2 overexpression on cell proliferation, MTT assay was used. Our results clearly indicated that NDRG2 overexpression decreased cell proliferation rate from third day and became more obviously thereafter compared to control groups (Figure1). The reduced proliferation of cancer cell lines after NDRG2 overexpression has been shown by other investigators.^9,13^ At molecular levels, it has been found that NDRG2 affects cell viability through interaction with tumor suppressor p53.^9^ In addition to p53, NDRG2 has been shown to affect cell viability through autophagy pathway.^15^

**Figure 2:**
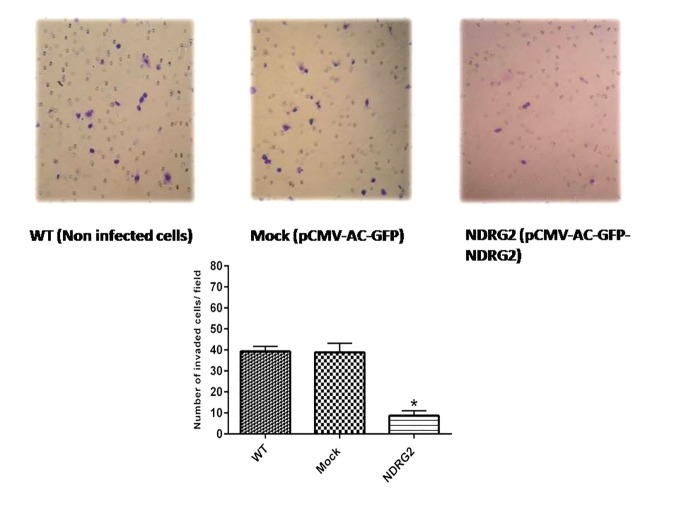
Invasion analysis of HCT116 cells treated using matrigel-coated transwells indicates a lower invasiveness of NDRG2 group cells than the other two groups. The assays were repeated thrice. The magnification of light microscope is 100×. *p<0.001.

**Figure 3:**
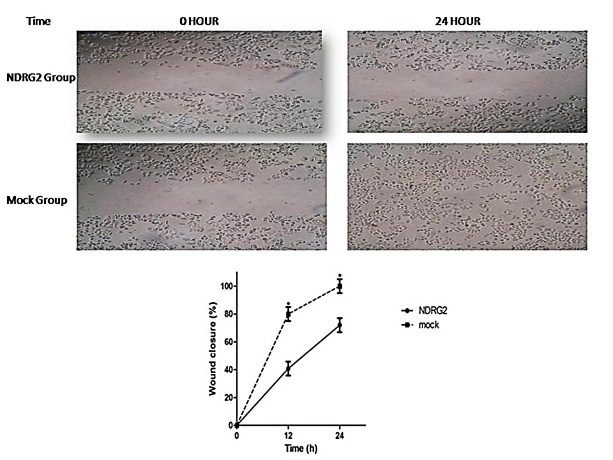
Wound healing assay for HCT116 cells. Photographs were taken at 40× magnification. The wound closure was quantified at 0h, and 24h post-wound by measuring the remaining unmigrated area using image J. *p<0.01.

The effect of NDRG2 overexpression on migration and invasion (matrigel coated transwells) revealed that forced expression of NDRG2 in HCT116 cells significantly suppressed their migration and invasion through the transwell membranes (P<0.001). Figure 2 indicates a representative of the number of traversed cells through matrigel coated transwells (data for migration assay not shown). Wound healing assay was used to determine the effect of NDRG2 overexpression on cell motility. After stable transfection, cells were stained at three interval times. This assay showed that after 24h, the NDRG2 group cells covered less than 73% of the wound, whereas mock group cells were able to close about 100% of the wound, indicating NDRG2 overexpression inhibits cell motility (Figure 3). Collectively, our results regarding migration and invasion ability of NDRG2 transfcted cells demonstrated a suppression in these activities, which is consistent with many prior publications,^13,16^ but not all.^17^

Gelatin zymography of the conditioned media of cells was used for analyzing secreted MMP-9 activity. Gelatin zymographic analysis revealed that secretion of gelatinolytic MMP-9 was significantly decreased in NDRG2 transfected cells (P<0.001; data not shown), indicating that suppressive activity of the cells on migration and invasion might partly be mediated through a reduction in MMP-9 activity. There are other mechanisms through which NDRG2 overexpression can suppress tumor invasion, notably the attenuation of NF-Kβ signaling,^16^ abrogation of laminin 232 pathway (a key pathway in tumor cell invasion, migration, and survival),^18^ and interaction with autophagy pathway.^15^

Our results in the current study are similar to our earlier findings on another colon cancer line SW48. HCT116 cell line has a mutation in Kras gene (codon 13).^14^ SW48 has no mutation in KRAS and considered a wild type in this respect. The *KRAS* is mutated in approximately 30%-50% of colorectal cancers. There are some targeted therapies in colon cancer that are administered to patients based on *KRAS* mutational status of tumor samples.^19^ In two separate studies, we confirmed that NDRG2 overexpression has anti-cancer effects in both KRAS mutated and nonmutated models. Whether manipulating NDRG2 can be therapeutic in a wide range of colon cancer patients independent of KRAS mutation needs more investigations.

## Conclusion

Our *in-vitro* model demonstrates that the NDRG2 overexpression attenuates several aspects of tumorigenesis including the proliferation and invasive potential of human colorectal cancer cell line HCT116, a widely used model for KRAS mutated colorectal cancer. Our data in combination with our prior publications on a KRAS mutated negative cell line point to NDRG2 as a potential therapeutic target for colorectal cancer.

## Acknowledgments

This work was financially supported by Shiraz Institute for Cancer Research (grant number: ICR 500). The authors declare no conflict of interest.

## Ethical Issues

Not applicable.

## Conflict of Interest

The authors declare no conflict of interests.
